# Genomic characteristics of *Lacticaseibacillus rhamnosus* strains isolated from blood

**DOI:** 10.1371/journal.pone.0335843

**Published:** 2025-10-31

**Authors:** Piotr Jarocki, Jan Sadurski, Martyna Siuda, Mateusz Romanowicz, Jacek Panek, Magdalena Frąc, Adam Waśko

**Affiliations:** 1 Department of Biotechnology, Microbiology and Human Nutrition, University of Life Sciences in Lublin, Lublin, Poland; 2 Department of Soil and Plant System, Laboratory of Molecular and Environmental Microbiology, Institute of Agrophysics, Polish Academy of Sciences, Lublin, Poland; Universidade dos Açores Departamento de Biologia: Universidade dos Acores Departamento de Biologia, PORTUGAL

## Abstract

*Lacticaseibacillus rhamnosus* is widely recognized for its health-promoting properties, which have led to its broad application in the production of food and dietary supplements. Nevertheless, although rare and typically limited to patients with underlying conditions, adverse effects have also been reported. In this study, we sequenced and characterized the genomes of seven *L. rhamnosus* strains isolated from blood. Using a hybrid approach that combined Illumina and Oxford Nanopore technologies, we obtained complete genomes ranging from 2.96 to 3.13 Mb, with a GC content of 46.7–46.8%. Comparative analyses with publicly available *L. rhamnosus* genomes revealed that these isolates were genetically related to strains from highly diverse origins, including plants, dairy products, dietary supplements, the gastrointestinal and genitourinary tracts, as well as blood and other clinical samples from geographically distant regions. Importantly, neither core genome multilocus sequence typing (cgMLST) nor prophage and CRISPR module analyses indicated similarity to the widely used probiotic strain *L. rhamnosus* GG. Gene-based analysis identified determinants associated with bacteriocin production, adhesion, health-promoting traits, and potential pathogenicity of the strains. Notably, several genes linked to probiotic functions also overlapped with virulence factors found in pathogenic microorganisms. These findings demonstrate the genomic diversity of *L. rhamnosus* blood isolates and highlight the dual role of certain genetic determinants, underlining the importance of careful strain-level evaluation when selecting *L. rhamnosus* strains for probiotic use.

## Introduction

*Lactobacillus rhamnosus*, recently reclassified as *Lacticaseibacillus rhamnosus*, is a widely studied bacterial species with important applications in the pharmaceutical and food industries [1,2]. Several strains, including GG, HN001, R0011, Oxy, Pen, E/N, and GR1, have demonstrated probiotic effects and are included in medical preparations with drug status [1,3–6]. Members of this species are gram-positive, homofermentative, non-spore-forming, and non-motile, and produce lactic acid from glucose [2,7,8]. As nomadic bacteria, *L. rhamnosus* inhabits diverse ecological niches, ranging from the human and animal gastrointestinal, genitourinary, and skin microbiota to fermented foods and environmental sources [2,8–10].

The beneficial effects of *L. rhamnosus* are mediated through multiple mechanisms, including intestinal colonization, inhibition of pathogenic microorganisms, and immune system modulation. Key contributing factors include the production of organic acids, bacteriocins, adhesion proteins, exopolysaccharides, and surface structures such as SpaCBA pili and lipoteichoic acids [11–19]. Clinically, *L. rhamnosus* strains have been associated with alleviating antibiotic-associated diarrhea, treating vaginal infections, improving oral and pulmonary health, and supporting immune function [5,20–22].

However, not all studies have reported consistent benefits. Some trials have found limited effects on antimicrobial-resistant colonization, microbiota restoration, and urinary tract infection prevention [23–26]. In addition, *L. rhamnosus* can pose risks in immunocompromised individuals, with documented cases of bacteremia, endocarditis, and other severe infections. These observations highlight that probiotic traits and pathogenicity are strain-specific, underscoring the need for precise strain-level identification in both research and clinical contexts [27–35].

While traditional methods for differentiating *L. casei* group bacteria were often labor-intensive and lacked reproducibility, advances in next-generation sequencing now allow comprehensive genome analyses for accurate identification and functional characterization [36–38]. In this study, we sequenced the complete genomes of seven blood-derived *L. rhamnosus* strains using hybrid sequencing method. The genomes were analyzed by core genome multilocus sequence typing (cgMLST) and subjected to functional genomic analyses to elucidate the unique features of these strains.

## Materials and methods

### Bacterial culture conditions and DNA isolation

*L. rhamnosus* strains (Table 1) were obtained from the Belgian Coordinated Collections of Microorganisms. Bacteria were cultured anaerobically in MRS broth (Difco) at 37°C. Genomic DNA was isolated and purified using the Genomic Mini AX Bacteria+ kit (A&A Biotechnology). The quality and concentration of genomic DNA were initially assessed using a NanoDrop spectrophotometer (Thermo Fisher Scientific) by measuring absorbance ratios at 260/280 nm and 260/230 nm. For more accurate quantification, DNA concentration was also measured using the Qubit 4.0 fluorometer (Thermo Fisher Scientific). DNA integrity was evaluated by electrophoresis on a 1% agarose gel stained with SimplySafe (Eurx) and visualized under UV light (Gel Doc XR + , Biorad).

**Table 1:** General features of complete genomic sequences obtained for the *L. rhamnosus* strains used in this study.

Strain	LMG 10768	LMG 23550	LMG 19716	LMG 19717	LMG 23277	LMG 23327	LMG 23551
**Isolation source**	Blood (Sweden)	Human with endocarditis, blood (UK)	Blood culture (Denmark)	Blood, terminal ileitis (Denmark)	Human, blood (Belgium)	Human, blood	Human with endocarditis, blood (UK)
**Genome size (bp)**	2926111	3127641	2918665	2922116	2988523	2958057	2964644
**Genome coverage**	465.5	390.6	221.1	583.9	136.5	133.9	246.1
**Contig numbers**	1	1	1	2	1	1	1
**Completeness (%)**	99.1	99.2	99.0	99.0	99.2	99.0	98.1
**DNA G + C (%)**	46.8	46.7	46.8	46.7	46.7	46.7	46.7
**Total genes**	2749	2949	2684	2771	2770	2773	2781
**Protein-coding genes**	2641	2817	2559	2655	2646	2667	2656
**rRNA genes**	15	15	15	15	15	15	15
**tRNA genes**	60	61	60	59	60	60	59
**ncRNA genes**	3	3	3	3	3	3	3
**Pseudogenes**	30	53	47	39	46	60	48
**Plasmids**	0	0	0	1	0	0	0
**Prophages**	2	5	1	3	3	4	4
**CRISPR arrays**	1	0	0	1	0	1	1
**GenBank accession**	CP136120.1	CP136119.1	CP136118.1	CP136116.1	CP136115.1	CP136114.1	CP136113.1

### Library preparation and genomic sequencing

The concentration of genomic DNA was measured prior to library preparation using PicoGreen reagent (Life Technologies) and a Tecan Infinite instrument.

Library preparation was performed using the NEBNext® Ultra™ II DNA Library Prep Kit for Illumina® (New England Biolabs, USA) following the manufacturer’s protocol. Briefly, 125 ng of genomic DNA was mechanically fragmented using a Covaris E210 sonicator (Covaris), then end-repaired and dA-tailed. NEBNext® adapters were ligated, and the libraries were purified with AMPure XP beads (Beckman Coulter). Six cycles of PCR amplification were performed using NEBNext® Ultra™ II Q5® Master Mix and Illumina-compatible TruSeq CD Indexes, followed by a second bead-based purification. Libraries were pooled by equal mass. The quality and size distribution of the final pooled library was assessed using an Agilent Bioanalyzer and quantified by qPCR. High-throughput sequencing was carried out using MiSeq Reagent Kit v3 (600 cycles) chemistry (Illumina) obtaining at least 50x coverage of the bacterial genome.

High-quality genomic DNA (500 ng per sample) was used as input for library preparation. Sequencing libraries were prepared using the Rapid Barcoding Kit (Oxford Nanopore Technologies, UK) according to the manufacturer’s protocol. Each bacterial isolate was uniquely barcoded, and the resulting libraries were pooled equimolarly. The final pooled library was loaded onto a MinION device equipped with a MinION Flow Cell R9.4.1. (Oxford Nanopore Technologies) for whole genome sequencing.

### Bioinformatics analysis

MiSeq reads were filtered using Cutadapt version 3.0 software [39]. Quality trimming was applied with a minimum Phred score of 25, and reads shorter than 15 bp after trimming were discarded. Quality control of sequencing data was performed with FastQC software [40]. Base calling for Oxford Nanopore sequences was carried out using Guppy version 6.1.2 (Oxford Nanopore) in high-accuracy mode with default Q-score settings. De novo assembly was performed using Unicycler version 0.4.7 [41] with default parameters. Genome assembly quality was assessed using QUAST [42] and CheckM [43].

The average nucleotide identity (ANI) of the obtained sequences was measured using the JSpecies Web Server [44] against selected reference genomes of *L. rhamnosus*, *L. casei*, *L. paracasei*, *L. chiayiensis*, and *L*. *zeae*. For cgMLST analysis, 615 publicly available *L. rhamnosus* genomic sequences were obtained from the NCBI Genome database using the NCBI Datasets tool. Genetic loci and alleles were identified, and a core gene set was determined. A schema was created using chewBBACA v3.3.2 [45] with default parameters: BLAST Score Ratio of 0.6, minimum length of 0, size threshold of 0.2, and translation table 11. A Prodigal training file was used for gene prediction. Loci present in at least 95% of the genomes were used for cgMLST analysis. A minimum spanning tree was generated using PHYLOViZ [46] and subsequently visualized in iTOL [47].

Prophage regions were identified with PHASTER using standard parameters, and classified into three groups (intact, questionable, and incomplete) based on the score values [48]. The presence of CRISPR/Cas modules was assessed using CRISPRCasFinder with the default settings [49]. Only sequences containing a complete set of *cas* genes were retained for further analysis. The COG evaluation was performed using the eggNOG-mapper software [50]. Potential probiotic characteristics and virulence factors were assessed using BLAST [51], ResFinder 4.6.0 [52], CARD [53], AMRFinderPlus [54], PathogenFinder [55], BAGEL4 [56], and ABRicate against Virulence Factor Database [57].

### Availability of data

The *L. rhamnosus* genome sequences have been deposited in the GenBank database under accession numbers: CP136113.1 - CP136120.1. All data generated or analyzed in this study are included in the article and its supplementary information files.

## Results and discussion

### General characteristics of the *L. rhamnosus* genome

In the present study, involving seven strains of *L. rhamnosus* isolated from blood (Table 1), genomes were obtained using sequencing with two technologies — Illumina (MiSeq) and Oxford Nanopore (MinION). Previous studies have concluded that this hybrid approach appears to be the optimal choice for obtaining complete bacterial genomes [58,59].

Using Illumina short-read sequencing, numerous reads ranging from 1,201,885 (for LMG 23550) to 2,080,408 (LMG 19716), with an average length of 227.0 to 245.2 bp, were obtained for each strain. The base call accuracy, expressed by the average Q-score and Q30%, was above 35 and 88%, respectively. The results obtained were characterized by acceptable quality [60]. The average GC content for all reads in individual samples ranged from 46.63 to 46.68%. De novo assembly of Illumina reads resulted in 47–76 contigs per genome. The assembly quality, expressed as the N50 value, varied between 136,078 and 338,799 bp, with L50 values of 4–6 contigs. The final coverage obtained for individual samples ranged from 89.1 to 174.8. Detailed results of Illumina sequencing (MiSeq) are shown in Table 2.

**Table 2:** Summary of sequencing results from the Miseq platform (Illumina), obtained using QUAST software.

Strain	LMG 10768	LMG 23550	LMG 19716	LMG 19717	LMG 23277	LMG 23327	LMG 23551
**Total bases**	380857055	278544378	510110592	416001163	323052358	341046233	291675488
**Number of reads**	1626164	1201885	2080408	1735350	1386667	1455906	1285001
**Mean read length**	234.2	231.8	245.2	239.7	233.0	234.3	227.0
**Mean Q**	35.7	35.8	35.7	35.8	35.8	35.6	35.8
**Q30%**	89.0	89.6	89.4	89.2	89.4	88.2	89.1
**Number of contigs**	65	76	52	73	64	47	74
**N50**	187530	136078	291847	220827	159007	338799	209859
**L50**	6	6	4	6	6	4	6
**N75**	129658	89039	116766	89931	83212	258243	115531
**L75**	10	12	8	11	13	7	11
**GC%**	46.73	46.66	46.73	46.63	46.68	46.65	46.65
**Mean coverage**	130.2	89.1	174.8	142.4	108.1	115.3	98.4

The application of Oxford Nanopore technology yielded reads, from 8,048 (LMG 23327) up to 190,157 (LMG 10768). The average read length for the samples tested ranged from 5,158.9 to 6,913.7 bp, with read N50 values between 10488 and 14472, indicating a substantial proportion of long reads suitable for resolving repetitive regions [61]. In the case of ONT, base call accuracy is usually much lower [62]; an average Q-score ranging from 11.9 to 12.5 was obtained for the tested samples. The approximate coverage for the tested strains varied from 18.6 (LMG 23327) to 441.5 (LMG 19717). The obtained results are included in Table 3.

**Table 3:** Summary of sequencing results from the MinION platform.

**Strain**	**LMG 10768**	**LMG 23550**	**LMG 19716**	**LMG 19717**	**LMG 23277**	**LMG 23327**	**LMG 23551**
**Total bases**	981246613	942813777	134991739	1290183975	84992644	55094906	437849943
**Number of reads**	190157	150805	24891	186614	16475	8048	83849
**Mean read length**	5160.2	6251.9	5423.3	6913.7	5158.9	6845.8	5221.9
**Median read length**	2708.0	3554.0	2822.0	4038.0	2401.0	3610.0	2871.0
**SD read length**	6465.3	7434.5	6780.2	8025.6	6815.0	8346.4	6408.9
**Read length N50**	10869.0	12485.0	11397.0	13500.0	11670.0	14472.0	10488.0
**Mean read quality**	11.9	11.9	11.9	11.9	11.9	11.9	12.0
**Median read quality**	12.5	12.5	12.5	12.5	12.5	12.5	12.6
**>Q7**	100.0%	100.0%	100.0%	100.0%	100.0%	100.0%	100.0%
**>Q10**	99.8%	99.9%	99.8%	99.9%	99.8%	99.6%	99.9%
**>Q12**	61.3%	60.8%	60.7%	60.6%	61.3%	61.2%	63.4%
**Mean coverage**	335.3	301.5	46.3	441.5	28.4	18.6	147.7

Finally, the reads obtained using both technologies were compiled using Unicycler software [46]. The final coverage for each genome ranged from 133.9 (LMG 23327) to 583.9 (LMG 19717) (Table 1). The obtained sequences met the basic minimum standards described by Riesco and Trujillo (2024) [63]. For all strains studied, full genomic sequences were obtained, with lengths ranging from 2,958,057 to 3,127,641 bp and GC content in the range of 46.7 to 46.8%. These values are consistent with previous literature reports describing genomes of strains belonging *to L. rhamnosus* [2,8].

In the analyzed genomes, 2,684 (LMG 19716) to 2,949 (LMG 23550) genes were identified, including sequences encoding rRNAs, tRNAs, ncRNAs, protein-coding genes, and pseudogenes (Table 1). Only for *L. rhamnosus* strain, LMG 19717 was a plasmid sequence of 46,439 bp obtained, in which 55 genes were identified. Similar plasmids were also detected in other *L. rhamnosus* strains, for example, hmr 1301 (98% sequence coverage, 99.98% sequence similarity), DM065 (84%, 99.54%), DM163 (84%, 99.56%), or PMC203 (81%, 99.54%). However, previous studies have shown that having plasmids is not a characteristic of all strains of this species [64].

### Phylogenetic analysis

In the next part of the work, a phylogenomic analysis of the obtained *L. rhamnosus* genomes was performed. Comparison of the studied sequences to selected *L. rhamnosus* genomes using the ANIb algorithm showed very high similarity, exceeding 96%, confirming the species affiliation of the analyzed strains. It is expected that for strains belonging to the same species, the ANI should be at least 95 to 96% [65]. For genomes from other species of the *L. casei* group, ANIb similarity was below 80%.

cgMLST was used to determine the phylogenetic status of the isolates at the strain level [66]. The first step was to identify all genes and determine the set of core genes necessary to create a cgMLST schema. Schema construction was performed using all available *L. rhamnosus* genomes (615) with complete genome, chromosome, scaffold, and contig status. In the analyzed genomes, 9,390 loci were identified, for which 78,757 alleles were detected. The most variable loci included 85 alleles for tyrosine-protein kinase CpsD, 81 for S8 family serine peptidase, and 78 for extracellular matrix-binding protein EbhA. In addition, 5,101 loci were present in only one variant. The general characteristics of the genes used and their alleles are shown in S1 Fig.

A cgMLST scheme was then developed, containing loci present in at least 95%, 99%, and 100% of the analyzed *L. rhamnosus* genomes (S2 Fig). Due to the low quality of some sequences, it is recommended that a set of core genes found in at least 95% of genomic sequences be used for further analyses [67]. For the 615 *L. rhamnosus* genomes, 1,813 loci were used to develop the cgMLST95 scheme. The list of core genes and variants present in the analyzed *L. rhamnosus* genomes is included in S1 Table. Based on the resulting scheme, the seven genomes of the studied strains were analyzed both individually and together alongside the 615 genomic sequences deposited for the *L. rhamnosus* species.

**Fig 1:**
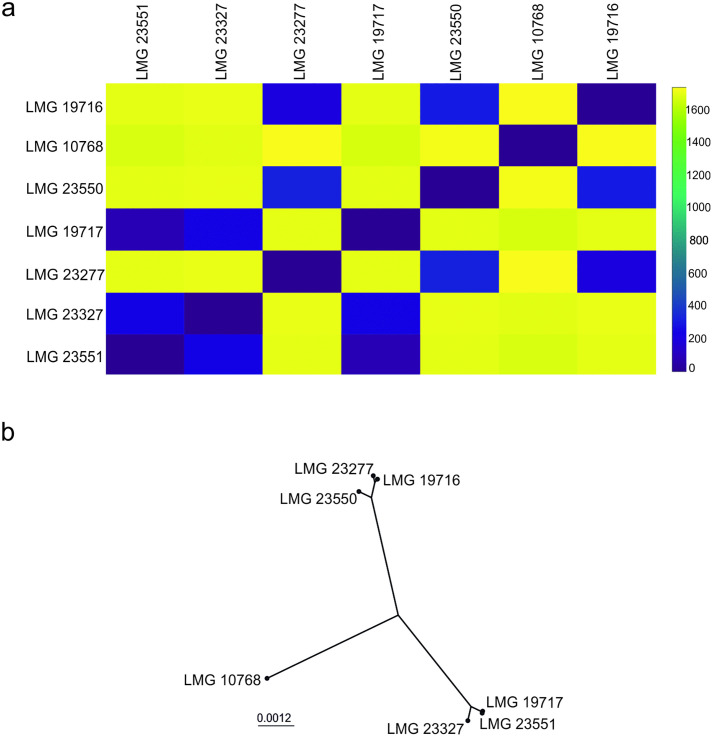
Analysis of the distances between seven *L. rhamnosus* strains based on core genome multilocus sequence typing (cgMLST) profiles obtained for 615 genomes. (A) Heatmap representing the allelic distance matrix for all samples in the dataset. The distances were computed by determining the number of allelic differences from the set of 1,778 core loci (loci that are not present in all samples are excluded from the calculation). (B) Core-genome neighbor-joining tree computed based on the multiple sequence alignment for the set of loci that constitute the core genome.

The analysis showed that among the coding sequences in the studied genomes, 1,808–1,813 gene loci were identified, based on which the cgMLST scheme was developed. This represented about 61 to 67% of all genes detected in the studied strains. Analysis of the seven tested strains indicated that the genomes obtained for isolates LMG 23551, LMG 23327, and LMG 19717; and LMG 23550, LMG 23277, and LMG 19716 were relatively similar (Fig 1).

**Fig 2:**
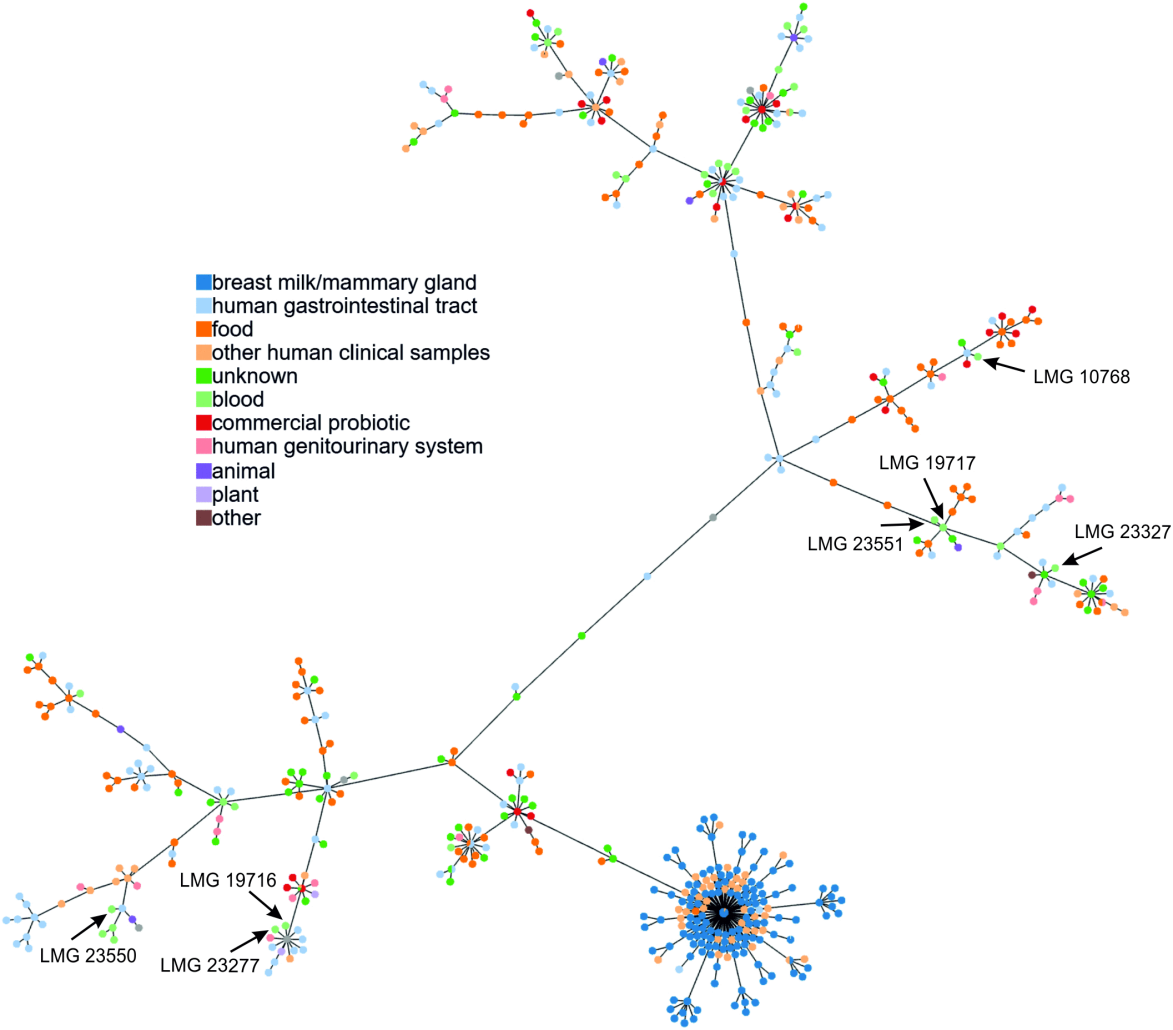
Minimum spanning tree of *L. rhamnosus* strains based on cgMLST allelic profiles, constructed using PHYLOViZ. The arrows indicate isolates derived from blood: LMG 10768, LMG 19716, LMG 19717, LMG 23277, LMG 23327, LMG 23550, and LMG 23551. The node colors represent the source of bacterial isolation.

Based on the allelic profile achieved for the 622 *L. rhamnosus* genomic sequences, an MST was constructed using PHYLOViZ software (Fig 2). The *L. rhamnosus* genomes most similar to the sequences of the tested strains were then identified, with both the source of isolation and geographic location taken into account (S2 Table and S3 Fig).

For strain LMG 23277, the most similar strains were bacteria isolated from the tongue (DM065 and DM163), stool (1001311H_170123_H11), and vaginal secretions (PMC203). Similar results were also obtained for strain LMG 19716, although the similarity to the above-mentioned strains was noticeably lower. Analysis of the genome of strain LMG 10768 revealed that the most similar isolates were ATCC 21052, LOCK900, and a strain derived from Egyptian cheese (CIRM-BIA 910). Slightly lower similarity was also observed with strains from a wide variety of sources, including commercial dietary supplements and pharmaceutical preparations. A very high degree of similarity was obtained for the two genomes presented in this paper (strains LMG 23551 and LMG 19717). These sequences were also similar to the genomes of isolates from dairy products from Georgia and Mongolia. The *L. rhamnosus* genomes most similar to the sequence obtained for strain LMG 23327 were those from bacteria derived from humans, including two strains isolated from urine. The last strain tested (LMG 23550) showed the highest homology with the INIA P344 isolate from infant feces (Spain), as well as several *L. rhamnosus* strains isolated from clinical or host-associated samples of intensive care patients (USA). Notably, this strain also showed similarity with three strains derived from blood [7].

In summary, the analysis of all *L. rhamnosus* genomes indicated that the studied strains showed high similarity to bacteria isolated from diverse sources, including the digestive tract, vagina, mouth, dairy products, and commercial probiotic supplements. It should also be emphasized that, due to the still insufficient number of genomic sequences from specific countries or regions, it was difficult to establish a definitive link between the studied strains and a particular geographical location.

As with the strains described by Nissilä et al. (2017), the genomic sequences obtained showed clear differences from that of the probiotic strain *L. rhamnosus* GG [7]. Due to its widespread use in health-promoting preparations, this strain has a global range. In some cases, correlation was observed with isolates from blood and other clinical samples, such as Lrh20, Lrh23, or Lrh30. These observations suggest the need for further research into the potential predisposition of these strains to colonize the human bloodstream.

### Prophage-like elements as a source of strain-specific sequences in the genomes of *L. rhamnosus*

Previous studies have shown the widespread occurrence and extreme diversity of prophage-like sequences in the genomes of bacteria belonging to the *L. casei* group [68]. The presence of such sequences may play an essential role in the bacterial host and may also be important in shaping the entire ecological niche. Notably, in gut bacteria, prophages may regulate the diversity of the gastrointestinal microbiota, indirectly influencing the overall physiological state of the intestine [69,70]. It has also been shown that spontaneous prophage induction (SPI) can have important roles in biofilm formation and horizontal gene transfer and may shape bacterial pathogenic traits [71]. Despite their mobile nature, the high diversity of these sequences can also be relevant for genotyping bacteria at the strain level [38].

In the obtained genomes, prophage sequence identification was carried out using PHASTER software. A total of 22 sequences were found, including three intact sequences (score above 90), 11 questionable sequences (score between 61 and 90), and eight incomplete sequences (score below 60). The length of the detected sequences ranged from 4.5 to 56.2 kb, with GC content ranging from 43.08 to 49.01%. Depending on length, the sequences contained between 7 and 67 genes (Table 4).

**Table 4:** General characterization of prophage sequences identified in the genomes of individual bacterial strains obtained in this study.

Strain	Region	Region Position	Completeness	Score	Region length [kb]	# Total proteins	GC %	Selected *L. rhamnosus* strains with similar prophage sequences (coverage/similarity)
**LMG 10768**	R3_1	776339-817727	questionable	89	41.3	57	44.62	Lrh20 (99/99.97), Lrh23 (99/99.97), NA 1–8 (87/99.27), AMC0714 (87/99.27), Lrh40 (87/99.27)
	R3_2	1872591-1920171	intact	150	47.5	60	44.51	1019 (42/92.21), Lr108 (40/87.04), Lrh5 (40/87.04), L35 (35/91.81), UMB8035A (40/84.83)
**LMG 23550**	R9_1	828486-871934	intact	150	43.4	63	44.1	Lrh31 (86/98.07), R19-3 (78/90.44), Lrh34 (75/91.48), NA 1–8 (43/100), LMG 24102 (55/97.03)
	R9_2	1119486-1175702	questionable	70	56.2	66	44.89	KF7 (73/97.34), LMG 23327 (70/94.10), LMG 23551 (68/94.10), NA 1–8 (67/99.87), LV108 (68/96.21)
	R9_3	1556056-1591861	incomplete	60	35.8	25	43.08	LMS2−1 (68/89.60), Lrh31 (64/91.27), GR-1 (64/91.06), DS22_11 (64/91.06), DS17_11 (64/91.09)
	R9_4	2042647-2047229	incomplete	30	4.5	8	49.01	DPC 7102 (100/100), LB C25 (100/100), RSI3 29 (100/100), INIA P344 (100/100), FAM 20558 (100/99.96)
	R9_5	3050998-3088435	questionable	90	37.4	36	45.07	VHProbi M12 (77/98.6), LMS2−1 (74/98.46), TOM.283 (71/98.110, PM4 (74,/99.11), 186_LRHA (69/97.15)
**LMG 19716**	R17_1	2850423-2867118	questionable	90	16.6	22	45.8	51B (90/99.96), LB (90/93.98) RSI3 (89/93.98), Lrh23 (71/96.15), Lrh28 (71/96.21), k32 (91/93.69)
**LMG 19717**	R18_1	784451-828861	intact	97	44.4	60	44.44	LMG 23551 (94/99.92), JCM 8130 (79/92.71), IDCC 3201 (70/91.49), Lrh30 (72/95.93), NA 1–8 (76/93.08)
	R18_2	987396-1001227	questionable	80	13.8	19	45.37	LMG 23551 (100/99.99), 1.0320 (100/99.99), LMG 23327 (100/99.91), Fmb14 (100/99.99)
	R18_3	1340530-1359064	incomplete	40	18.5	19	46.38	1.0320 (100/99.99), LMG 23551 (100/99.98), LR5 (100/99.98), LMG 23327 (100/99.98), Fmb14 (100/99.98)
**LMG 23277**	R23_1	838430-884645	questionable	89	46.2	60	44.94	PMC203 (95/100.00), YGRT94 (95/100.00), DM163 (95/100.00), AMC0712 (95/100.00), cek-R1 (95/100.00)
	R23_2	1048793-1063311	incomplete	20	14.5	18	43.63	PMC203 (100/99.99), YGRT94 (100/99.99), DM163 (100/99.99), AMC0712 (100/99.99), cek-R1 (100/99.99)
	R23_3	1490792-1497481	incomplete	40	6.6	7	44.84	FUA3185 (68/100.00), cek-R1 (68/100.00), Lrh40 (68/100.00), (68/100.00), LMG 23550 (68/100.00)
**LMG 23327**	R24_1	948455-962297	questionable	80	13.8	19	45.37	UMB1351A (100/100.00), UMB0004 (100/100.00), Lrh34 (100/100.00), Lrh11 (100/100.00)
	R24_2	1044833-1089324	questionable	77	44.4	67	44.67	319_LRHA (100/99.9), 784_LRHA (100/99.98), LRHMDP3 (99/99.94), LRHMDP2 (99/99.94)
	R24_3	1342719-1361253	incomplete	40	18.5	18	46.39	LR5 (100/99.99), Fmb14 (100/99.99), 1.0320 (100/99.99), LMG 23551 (100/99.98), LMG 19717 (100/99.98)
	R24_4	1789871-1841376	questionable	81	51.5	55	44.76	lbd330 (76/100.00), lbr108 (76/100.00), LMG 23551 (51/100.00), LMG 19717 (50/100.00)
**LMG 23551**	R26_1	787692-830897	questionable	76	43.2	57	44.27	LMG 19717 (90/99.17), PM4 (66/94.34), JCM 8130 (76/92.69), KF7 (69/93.59), NA 1–8 (73/93.23)
	R26_2	989432-1003263	questionable	80	13.8	19	45.38	lbd85−1 (100/99.99), 1.0320 (100/99.99), LMG 23327 (100/99.91), LMG 19717 (100/99.99), 116 (100/99.99)
	R26_3	1085879-1127562	incomplete	60	41.6	64	44.56	Lrh11 (74/97.74), KF7 (89/91.99), Fmb14 (80/97.74), LMG 23327 (85/94.28), 308 (78/92.99)
	R26_4	1382567-1401101	incomplete	40	18.5	19	46.38	LR5 (100/99.99), Fmb14 (100/99.99), 1.0320 (100/99.99), LMG 23551 (100/99.98), LMG 19717 (100/99.98)

For strain LMG 10768, two prophage sequences were identified, one of which (R3_1) was almost identical to sequences from blood isolates Lrh20 and Lrh23, suggesting possible associations between certain prophages and strains of clinical origin. In contrast, the second sequence (R3_2) appeared to be strain-specific, with only fragmentary homologs in other genomes. The chromosome of LMG 23550, was particularly rich in prophage sequences, which represented nearly 6% of the total genome. One sequence, only 4.5 kb long and encoding eight proteins (including an IS3 family transposase), was found in many strains of both *L. rhamnosus* and in closely related species. The remaining prophages displayed only partial similarity to those found in other solates, indicating that analyzed strain harbors unique mobile elements.

Only one prophage-like sequence was identified in *L. rhamnosus* LMG 19716. Similar prophages with 91–94% similarity were present in the genomes of *L. rhamnosus* (k32, 51B, LB, RSI3) and *L. casei* (FBL6) strains. This suggests that certain phage elements may circulate within closely related species. In the *L. rhamnosus* LMG 19717 genome, three prophages were detected. Two (R18_2 and R18_3) were widely distributed among *L. rhamnosus* strains, whereas the third sequence (R18_1) contained fragments that were unique to this strain. The most similar sequence, with 99.92% similarity and 94% sequence coverage, was found in the *L. rhamnosus* LMG 23551 genome obtained in this study. In another strain (LMG 23277), three prophages were identified that were widespread across the species but often displayed incomplete coverage, highlighting structural variation even in conserved sequences. Finally, the last two strains (LMG 23327 and LMG 23551) carried multiple prophages, some of which (e.g., R24_1 and R26_2) were broadly distributed across *L. rhamnosus* and *L. paracasei* strains from diverse environments, whereas others were more restricted (Table 4).

Overall, the analysis of prophage-like sequences confirmed the broad occurrence of such elements in *L. rhamnosus* genomes, comprising both universal and strain-specific sequences. This variability not only highlights the dynamic nature of the *L. rhamnosus* mobilome but also constitutes a source of specific sequences enabling the identification of individual isolates at the strain level [38,68,72].

### Characterization of CRISPR-Cas systems in analyzed strains

CRISPR-Cas systems are also essential components of bacterial genomes. Given the high variability of motifs referred to as spacers, these sequences can be used for genotyping bacteria at the strain level. Bacterial identification methods based on CRISPR loci are especially relevant for the specific detection of pathogens in clinical samples and food [73–75]. However, a limitation of this approach is that the presence of CRISPR motifs is not universal. It is estimated that CRISPR sequences are observed in only about 40% of bacterial genomes [76].

In the present study, complete CRISPR modules categorized as the CAS-Type IIA subtype were detected in four of the seven genomes examined. For strains LMG 23327, LMG 23551, LMG 19717, and LMG 10768, 28, 37, 27, and 40 spacers were identified, respectively, with identical repeat sequences of 36 nucleotides in length located between them. Genes encoding CRISPR-associated (Cas) proteins — Cas1, Cas2, Cas9, and Csn2 — were also detected. A detailed analysis of CRISPR modules *in L. rhamnosus* genomes revealed the presence of similar sequences in other strains. For three of the four strains tested with the CRISPR module, some spacer sequences were strain-specific (S3 Table).

An alignment of the CRISPR module obtained for strain LMG 10768 with *L. rhamnosus* genomes showed the highest similarity to clinical isolate 186_LRHA. In the genome of this strain, 59 spacer sequences were detected; however, compared to LMG 10768, spacer sequences 5, 39, and 40 were missing. The DSM 14870 and TK-F8B strains also shared high similarity, each with 27 identical spacers. Notably, the last spacer was identified in plasmid sequences of *L. plantarum* and *L. kefiri*.

For LMG 23327, six strains had identical CRISPR modules (QAULRN2, RAB2019A, Fmb14, 1001095st1_F3_1001254B_151014, 1001095st1_F6_1001095A_150126, and UW_DM_LACCAS2_1), all sharing 28 spacers. In addition, CRISPR sequences with very high similarity were also detected in many other *L. rhamnosus* genomes. Individual spacer analysis revealed that spacer 5 was present in phage Lrm1, spacer 26 in *L. plantarum* plasmids, while spacer 27 was identified in the genomes of phages T25, R9.3, C3.1, and C4.1. Spacer 28 was commonly found in many phages of the *L. casei* group, including BH1, C3.1, C4.1, R3.1, R9.2, R29.1, R18.1, and R26.14, as well as in several sequences of *Caudoviricetes* sp. and *Siphoviridae* sp. obtained from metagenomic studies [77].

The CRISPR array obtained from the genome of LMG 19717 contained 27 spacer sequences, 26 of which were also found in the genomes of strains LMG 23551, UMB13151A, and LR5. Some spacer sequences were further identified in *L. rhamnosus* strains SD4, SD11, Fmb14, UMB0004, WHH1155, LMG 23327, and others. In addition, some sequences, specifically spacers 4, 26, and 27, were detected in mobile elements, including *L. plantarum* plasmids and *L. casei* group phages [68,78].

For the final strain (LMG 23551), BLAST analysis showed highest similarity to UMB131A and LMG 19717, with 26 identical spacers out of the 37. Similar sequences were also detected in LR5, Fmb14, UMB0004, SD4, SD11, LMG 23327, and WHH1155, originating from various sources (dairy-fermented products, feces, saliva, and blood). Several spacers have been identified in the plasmids of species formerly classified within the genus *Lactobacillus,* as well as in *Pediococcus* and *Lactococcus*. Notably, spacers 4 and 29 were observed in the phage sequences of Lrm1 and BH1 [78,79].

In conclusion, CRISPR modules, particularly spacer sequences, appear to be essential components of bacterial genomes, aiding in the reconstruction of the genetic history of individual bacterial strains. The identification of similar sequences across different isolates suggests a close evolutionary relationship and a shared lineage. Furthermore, the enormous diversity of these motifs may facilitate the development of strain-specific molecular probes for bacterial identification. Among the strains studied, in some cases, genomes were identified with an identical set of spacers, indicating a possible close genetic relationship. In addition, conserved sequences were observed across the tested strains. Interestingly, the complete set of spacer sequences identified in this study was absent from the CRISPR module of the *L. rhamnosus* GG genome. This finding suggests that, despite its widespread distribution, *L. rhamnosus* GG is not closely related to the blood-derived isolates examined. However, spacer sequences originating from the *L. rhamnosus* GG genome were present in the prophage sequences identified in the genomes of strains LMG 23277 and LMG 23327.

### Functional analysis of genomic sequences

**Fig 3:**
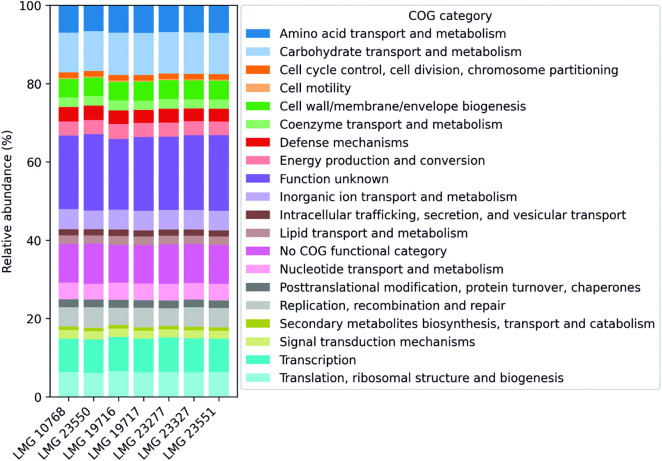
Relative abundance of COG (Clusters of Orthologous Groups) categories across the analyzed genomes. Each COG category is represented by a distinct color, enabling comparison of functional category distributions among genomes.

The genes identified in the *L. rhamnosus* genomes were classified into 19 functional categories of clusters of orthologous groups (COGs) (Fig 3 and S4 Table). For all strains tested, approximately 10% of the genes were not assigned to any category. Among the genes with a specific COG class, the largest group (about 10.2–10.8%) consisted of sequences encoding proteins related to the transport and metabolism of carbohydrates, amino acids (6.6–7%), transcription (8.5–8.8%), and translation (6–6.5%). Nearly 20% of the genes were sequences classified in the category for genes of undetermined function. This emphasizes the need for further research to better understand the identified genes. Notably, the number of genes in each COG category was fairly similar across the strains studied. The low variation between isolates may reflect similar adaptive strategies for survival in a specific ecological niche [8].

Analysis performed with ResFinder 4.6.0, CARD, and AMRFinderPlus software did not indicate the presence of genes associated with antibiotic resistance. Only in the case of *L. rhamnosus* LMG 10768 was a sequence encoding the MFS transporter detected — a protein responsible for transporting ions, sugar phosphates, drugs, neurotransmitters, nucleosides, amino acids, and peptides across cytoplasmic or internal membranes [80,81]. This finding is important for potential antibiotic therapy in bacteremia caused by *L. rhamnosus* strains. Moreover, PathogenFinder, available through the Center for Genomic Epidemiology resources, indicated that regardless of the isolation source, the strains studied cannot be classified as human pathogens [55]. Similarly, screening against the Virulence Factor Database (VFDB) using ABRicate did not reveal the presence of known virulence determinants [57]. In line with previous studies involving clinical *L. rhamnosus* strains, it remains difficult to definitively identify the genes that determine the ability of these isolates to cause bacteremia. Based on scientific reports, it can be speculated that factors such as adhesion-related proteins, modified EPS clusters, pilus genes, or the phenomenon of SPI may play a role, among others [7,35,71,82]. However, these elements provide physiologically relevant functions that facilitate colonization of the human gastrointestinal tract and are present in many strains that are considered as safe.

Comparative analysis revealed the presence of genes encoding sortases and the proteins they recognize, which contain the LPXTG motif (S5 Table). These proteins are considered important adhesion factors that affect the ability of bacteria to bind to intestinal epithelial cells [83]. Studies have also shown that these proteins are very important pathogenicity factors [84,85]. Thus, what may appear to be a beneficial, health-promoting feature could instead contribute to infection, particularly in patients with health issues, for whom probiotic use should be limited [27,86]. Similar to the strains described by Nadkarni et al. (2014) and Nissilä et al. (2017), *spaFED* clusters encoding pilus proteins were detected in the analyzed genomes [7,82]. Their role in bacterial adhesion has been well documented [87,88]. The second pilus module (*spaCBA*) was present only in *L. rhamnosus* strain LMG 19717 and was interestingly located on a plasmid (CP136117.1). Analysis showed that *spaCBA* is less common among *L. rhamnosus* strains, although a similar sequence was also observed in the genomes and plasmids of *L. paracasei* [89]. Due to the presence of mobile elements near the *spaCBA* genes, it is possible that this module was acquired via horizontal gene transfer.

The production of EPS is another crucial factor that influences adhesion properties and biofilm formation. In the genomes of the strains studied, many sequences related to EPS biosynthesis were detected, with their roles in the production of these compounds already established [14]. In all genomes examined, at least one complete cluster encoding genes related to EPS production was present (S5 Table). It should be noted that these modules exhibit high variability. In some cases, genes encoding proteins with specific functions in EPS synthesis showed no sequence similarity to genes previously described for *L. rhamnosus* GG. This may be due to the presence of mobile elements within these sequences, such as transposase enzymes [7].

Genetic determinants responsible for the synthesis of bacteriocins were the final element investigated in the *L. rhamnosus* genomes. These proteins exhibit bacteriostatic properties, making them relevant for the direct competition between *L. rhamnosus* strains and other bacteria within the same ecological niche [90,91]. Previous studies have shown that bacteria of this species can synthesize bacteriocins such as carnocine CP52, enterocin X chain beta, and class II bacteriocins with a double-glycine leader peptide [92]. Similar genetic clusters were also detected in all *L. rhamnosus* genomes analyzed in this study (S5 Table).

## Conclusions

In this study, we obtained the complete genome sequences of seven *L. rhamnosus* blood isolates using a hybrid short-read/long-read sequencing approach. Hybrid sequencing is an optimal strategy for obtaining complete bacterial genomes, enabling precise characterization and identification of specific strains relevant to industry or medicine. Comparative analysis using cgMLST demonstrated phylogenetic relationships with previously characterized *L. rhamnosus* strains, confirming the utility of this method for strain-level differentiation of *L. rhamnosus*. Based on the results, it can be concluded that the strains studied do not show a clear similarity with only clinical isolates. In addition, they are not closely related to commercial strains (such as *L. rhamnosus* GG), which are used in dietary supplements and as food additives. The analysis revealed a wide diversity of strains in terms of both the source of isolation and geographic origin, which exhibited similarity to the *L. rhamnosus* strains studied. Similar conclusions can be drawn from analyses based on prophage sequences and CRISPR arrays. Given the very high genetic diversity, these modules appear to be a valuable complement to genomic analyses, such as cgMLST. Genomic analysis also identified numerous genes associated with traits commonly linked to probiotics. Some of these genes are also present in pathogenic bacteria and have been implicated in microbial virulence. These findings emphasize that the mere presence of such genes in a genome does not necessarily indicate beneficial effects and should be interpreted carefully when characterizing bacterial strains.

## Supporting information

S1 Fig cgMLST schema data summary, including. (A) Distribution of the number of alleles for all loci, (B) Distribution of the allele mode size (most frequent allele size) for all loci, and (C) Total number of alleles and the minimum, maximum, and median allele size per locus. Loci and alleles were identified in 615 *L. rhamnosus* genomes (complete genomes, chromosomes, scaffolds, and contigs).
(TIF)

S2 Fig Analysis of the loci number in cgMLST, assuming that a given gene is present in 95, 99, and 100% of the analyzed genomes. The observation was performed using 615 *L. rhamnosus* genomic sequences (including complete genomes, chromosomes, scaffolds, and contigs).
(TIF)

S3 Fig Minimum spanning tree of *L. rhamnosus* strains based on cgMLST allele profiles. Distances were calculated with cgmlst/dists, and the MST was generated using NetworkX (Kruskal’s algorithm). The resulting tree was exported in Newick format and visualized with iTOL. Concentric circles around strains indicate metadata: the inner circle shows geographic origin, and the outer circle shows the isolation source, both encoded with colors as specified in the legend. Strains analyzed in this study are highlighted in green.
(PDF)

S1 Table Occurrence of individual genes and alleles in the 615 *L. rhamnosus* genomes. Loci present in at least 95% of the analyzed genomic sequences were used to construct the cgMLST scheme.
(XLSX)

S2 Table Distance matrices for all tested strains, constructed from a node selection of the cgMLST *L. rhamnosus* dataset. (XLSX)

S3 Table Basic information on CRISPR/Cas sequences identified in the genomes of *L. rhamnosus* strains LMG 10768, LMG 19717, LMG 23327 and LMG 23551. (XLSX)

S4 Table Functional study of *L. rhamnosus* genome sequences, presenting the distribution of genes within individual COG categories. (XLSX)

S5 Table Analysis of the occurrence of genes encoding proteins related to adhesion, production of exopolysaccharides, and bacteriocins in the genomes of the *L. rhamnosus* strains studied. (XLSX)

## References

[1] Capurso L. Thirty Years of *Lactobacillus rhamnosus* GG: A Review. J Clin Gastroenterol. 2019;53(1):S1–41. doi: 10.1097/MCG.0000000000001170 30741841

[2] Zheng J, Wittouck S, Salvetti E, Franz CMAP, Harris HMB, Mattarelli P, et al. A taxonomic note on the genus *Lactobacillus*: Description of 23 novel genera, emended description of the genus *Lactobacillus* Beijerinck 1901, and union of Lactobacillaceae and Leuconostocaceae. Int J Syst Evol Microbiol. 2020;70(4):2782–858. doi: 10.1099/ijsem.0.004107 32293557

[3] Miao X, Jiang Y, Kong D, Wu Z, Liu H, Ye X, et al. *Lactobacillus rhamnosus* HN001 Ameliorates BEZ235-Induced Intestinal Dysbiosis and Prolongs Cardiac Transplant Survival. Microbiol Spectr. 2022;10(4):e0079422. doi: 10.1128/spectrum.00794-22 35862958 PMC9430965

[4] Foster LM, Tompkins TA, Dahl WJ. A comprehensive post-market review of studies on a probiotic product containing *Lactobacillus helveticus* R0052 and *Lactobacillus rhamnosus* R0011. Benef Microbes. 2011;2(4):319–34. doi: 10.3920/BM2011.0032 22146691

[5] Ruszczyński M, Radzikowski A, Szajewska H. Clinical trial: effectiveness of *Lactobacillus rhamnosus* (strains E/N, Oxy and Pen) in the prevention of antibiotic-associated diarrhoea in children. Aliment Pharmacol Ther. 2008;28(1):154–61. doi: 10.1111/j.1365-2036.2008.03714.x 18410562

[6] Nader-Macías MEF, De Gregorio PR, Silva JA. Probiotic lactobacilli in formulas and hygiene products for the health of the urogenital tract. Pharmacol Res Perspect. 2021;9(5):e00787. doi: 10.1002/prp2.787 34609059 PMC8491456

[7] Nissilä E, Douillard FP, Ritari J, Paulin L, Järvinen HM, Rasinkangas P, et al. Genotypic and phenotypic diversity of *Lactobacillus rhamnosus* clinical isolates, their comparison with strain GG and their recognition by complement system. PLoS One. 2017;12(5):e0176739. doi: 10.1371/journal.pone.0176739 28493885 PMC5426626

[8] Douillard FP, Ribbera A, Kant R, Pietilä TE, Järvinen HM, Messing M, et al. Comparative genomic and functional analysis of 100 *Lactobacillus rhamnosus* strains and their comparison with strain GG. PLoS Genet. 2013;9(8):e1003683. doi: 10.1371/journal.pgen.1003683 23966868 PMC3744422

[9] Ceapa C, Lambert J, van Limpt K, Wels M, Smokvina T, Knol J, et al. Correlation of *Lactobacillus rhamnosus* Genotypes and Carbohydrate Utilization Signatures Determined by Phenotype Profiling. Appl Environ Microbiol. 2015;81(16):5458–70. doi: 10.1128/AEM.00851-15 26048937 PMC4510185

[10] Kant R, Rintahaka J, Yu X, Sigvart-Mattila P, Paulin L, Mecklin J-P, et al. A comparative pan-genome perspective of niche-adaptable cell-surface protein phenotypes in *Lactobacillus rhamnosus*. PLoS One. 2014;9(7):e102762. doi: 10.1371/journal.pone.0102762 25032833 PMC4102537

[11] Corr SC, Hill C, Gahan CGM. Understanding the mechanisms by which probiotics inhibit gastrointestinal pathogens. Adv Food Nutr Res. 2009;56:1–15. doi: 10.1016/S1043-4526(08)00601-3 19389605

[12] Mantegazza C, Molinari P, D’Auria E, Sonnino M, Morelli L, Zuccotti GV. Probiotics and antibiotic-associated diarrhea in children: A review and new evidence on *Lactobacillus rhamnosus* GG during and after antibiotic treatment. Pharmacol Res. 2018;128:63–72. doi: 10.1016/j.phrs.2017.08.001 28827186

[13] Kankainen M, Paulin L, Tynkkynen S, von Ossowski I, Reunanen J, Partanen P, et al. Comparative genomic analysis of *Lactobacillus rhamnosus* GG reveals pili containing a human- mucus binding protein. Proc Natl Acad Sci U S A. 2009;106(40):17193–8. doi: 10.1073/pnas.0908876106 19805152 PMC2746127

[14] Lebeer S, Verhoeven TLA, Francius G, Schoofs G, Lambrichts I, Dufrêne Y, et al. Identification of a Gene Cluster for the Biosynthesis of a Long, Galactose-Rich Exopolysaccharide in *Lactobacillus rhamnosus* GG and Functional Analysis of the Priming Glycosyltransferase. Appl Environ Microbiol. 2009;75(11):3554–63. doi: 10.1128/AEM.02919-08 19346339 PMC2687306

[15] Makras L, Triantafyllou V, Fayol-Messaoudi D, Adriany T, Zoumpopoulou G, Tsakalidou E, et al. Kinetic analysis of the antibacterial activity of probiotic lactobacilli towards *Salmonella enterica* serovar Typhimurium reveals a role for lactic acid and other inhibitory compounds. Res Microbiol. 2006;157(3):241–7. doi: 10.1016/j.resmic.2005.09.002 16266797

[16] Doron S, Snydman DR, Gorbach SL. *Lactobacillus* GG: bacteriology and clinical applications. Gastroenterol Clin North Am. 2005;34(3):483–98, ix. doi: 10.1016/j.gtc.2005.05.011 16084309

[17] Segers ME, Lebeer S. Towards a better understanding of Lactobacillus rhamnosus GG--host interactions. Microb Cell Fact. 2014;13 Suppl 1(Suppl 1):S7. doi: 10.1186/1475-2859-13-S1-S7 25186587 PMC4155824

[18] Lebeer S, Claes I, Tytgat HLP, Verhoeven TLA, Marien E, von Ossowski I, et al. Functional analysis of *Lactobacillus rhamnosus* GG pili in relation to adhesion and immunomodulatory interactions with intestinal epithelial cells. Appl Environ Microbiol. 2012;78(1):185–93. doi: 10.1128/AEM.06192-11 22020518 PMC3255643

[19] Claes IJJ, Segers ME, Verhoeven TLA, Dusselier M, Sels BF, De Keersmaecker SCJ, et al. *Lipoteichoic* acid is an important microbe-associated molecular pattern of *Lactobacillus rhamnosus* GG. Microb Cell Fact. 2012;11:161. doi: 10.1186/1475-2859-11-161 23241240 PMC3537616

[20] Liu P, Lu Y, Li R, Chen X. Use of probiotic lactobacilli in the treatment of vaginal infections: *In vitro* and *in vivo* investigations. Front Cell Infect Microbiol. 2023;13:1153894. doi: 10.3389/fcimb.2023.1153894 37077531 PMC10106725

[21] Du T, Lei A, Zhang N, Zhu C. The Beneficial Role of Probiotic *Lactobacillus* in Respiratory Diseases. Front Immunol. 2022;13:908010. doi: 10.3389/fimmu.2022.908010 35711436 PMC9194447

[22] Alanzi A, Honkala S, Honkala E, Varghese A, Tolvanen M, Söderling E. Effect of *Lactobacillus rhamnosus* and *Bifidobacterium lactis* on gingival health, dental plaque, and periodontopathogens in adolescents: a randomised placebo-controlled clinical trial. Benef Microbes. 2018;9(4):593–602. doi: 10.3920/BM2017.0139 29633646

[23] Rauseo AM, Hink T, Reske KA, Seiler SM, Bommarito KM, Fraser VJ, et al. A randomized controlled trial of *Lactobacillus rhamnosus* GG on antimicrobial-resistant organism colonization. Infect Control Hosp Epidemiol. 2022;43(2):167–73. doi: 10.1017/ice.2021.94 33820576

[24] Suez J, Zmora N, Zilberman-Schapira G, Mor U, Dori-Bachash M, Bashiardes S, et al. Post-Antibiotic Gut Mucosal Microbiome Reconstitution Is Impaired by Probiotics and Improved by Autologous FMT. Cell. 2018;174(6):1406-1423.e16. doi: 10.1016/j.cell.2018.08.047 30193113

[25] Colodner R, Edelstein H, Chazan B, Raz R. Vaginal colonization by orally administered *Lactobacillus rhamnosus* GG. Isr Med Assoc J. 2003;5(11):767–9. 14650098

[26] Suez J, Zmora N, Segal E, Elinav E. The pros, cons, and many unknowns of probiotics. Nat Med. 2019;25(5):716–29. doi: 10.1038/s41591-019-0439-x 31061539

[27] Yelin I, Flett KB, Merakou C, Mehrotra P, Stam J, Snesrud E, et al. Genomic and epidemiological evidence of bacterial transmission from probiotic capsule to blood in ICU patients. Nat Med. 2019;25(11):1728–32. doi: 10.1038/s41591-019-0626-9 31700189 PMC6980696

[28] Kochan P, Chmielarczyk A, Szymaniak L, Brykczynski M, Galant K, Zych A, et al. *Lactobacillus rhamnosus* administration causes sepsis in a cardiosurgical patient--is the time right to revise probiotic safety guidelines? Clin Microbiol Infect. 2011;17(10):1589–92. doi: 10.1111/j.1469-0691.2011.03614.x 21848974

[29] D’Agostin M, Squillaci D, Lazzerini M, Barbi E, Wijers L, Da Lozzo P. Invasive Infections Associated with the Use of Probiotics in Children: A Systematic Review. Children (Basel). 2021;8(10):924. doi: 10.3390/children8100924 34682189 PMC8534463

[30] Chiang M-C, Chen C-L, Feng Y, Chen C-C, Lien R, Chiu C-H. *Lactobacillus rhamnosus* sepsis associated with probiotic therapy in an extremely preterm infant: Pathogenesis and a review for clinicians. J Microbiol Immunol Infect. 2021;54(4):575–80. doi: 10.1016/j.jmii.2020.03.029 32307246

[31] Aaron JG, Sobieszczyk ME, Weiner SD, Whittier S, Lowy FD. *Lactobacillus rhamnosus* Endocarditis After Upper Endoscopy. Open Forum Infect Dis. 2017;4(2):ofx085. doi: 10.1093/ofid/ofx085 28695143 PMC5499731

[32] Tang Q, Hao Y, Wang L, Lu C, Li M, Si Z, et al. Characterization of a bacterial strain *Lactobacillus paracasei* LP10266 recovered from an endocarditis patient in Shandong, China. BMC Microbiol. 2021;21(1):183. doi: 10.1186/s12866-021-02253-8 34134621 PMC8210379

[33] Conen A, Zimmerer S, Trampuz A, Frei R, Battegay M, Elzi L. A pain in the neck: probiotics for ulcerative colitis. Ann Intern Med. 2009;151(12):895–7. doi: 10.7326/0003-4819-151-12-200912150-00020 20008769

[34] Costa RL, Moreira J, Lorenzo A, Lamas CC. Infectious complications following probiotic ingestion: a potentially underestimated problem? A systematic review of reports and case series. BMC Complement Altern Med. 2018;18(1):329. doi: 10.1186/s12906-018-2394-3 30541524 PMC6292120

[35] Rossi F, Amadoro C, Colavita G. Members of the Lactobacillus Genus Complex (LGC) as Opportunistic Pathogens: A Review. Microorganisms. 2019;7(5):126. doi: 10.3390/microorganisms7050126 31083452 PMC6560513

[36] Jarocki P, Komoń-Janczara E, Glibowska A, Dworniczak M, Pytka M, Korzeniowska-Kowal A, et al. Molecular Routes to Specific Identification of the *Lactobacillus Casei* Group at the Species, Subspecies and Strain Level. Int J Mol Sci. 2020;21(8):2694. doi: 10.3390/ijms21082694 32294944 PMC7216162

[37] Huang C-H, Li S-W, Huang L, Watanabe K. Identification and Classification for the *Lactobacillus casei* Group. Front Microbiol. 2018;9. doi: 10.3389/fmicb.2018.01974 PMC6113361 30186277

[38] Uelze L, Grützke J, Borowiak M, Hammerl JA, Juraschek K, Deneke C, et al. Typing methods based on whole genome sequencing data. One Health Outlook. 2020;2:3. doi: 10.1186/s42522-020-0010-1 33829127 PMC7993478

[39] Martin M. Cutadapt removes adapter sequences from high-throughput sequencing reads. EMBnet J. 2011;17(1):10–2.

[40] Andrews S. FastQC: A quality control tool for high throughput sequence data [Online]. 2010. http://www.bioinformatics.babraham.ac.uk/projects/fastqc/

[41] Wick RR, Judd LM, Gorrie CL, Holt KE. Unicycler: Resolving bacterial genome assemblies from short and long sequencing reads. PLoS Comput Biol. 2017;13(6):e1005595. doi: 10.1371/journal.pcbi.1005595 28594827 PMC5481147

[42] Gurevich A, Saveliev V, Vyahhi N, Tesler G. QUAST: quality assessment tool for genome assemblies. Bioinformatics. 2013;29(8):1072–5. doi: 10.1093/bioinformatics/btt086 23422339 PMC3624806

[43] Parks DH, Imelfort M, Skennerton CT, Hugenholtz P, Tyson GW. CheckM: assessing the quality of microbial genomes recovered from isolates, single cells, and metagenomes. Genome Res. 2015;25(7):1043–55. doi: 10.1101/gr.186072.114 25977477 PMC4484387

[44] Richter M, Rosselló-Móra R, Oliver Glöckner F, Peplies J. JSpeciesWS: a web server for prokaryotic species circumscription based on pairwise genome comparison. Bioinformatics. 2016;32(6):929–31. doi: 10.1093/bioinformatics/btv681 26576653 PMC5939971

[45] Silva M, Machado MP, Silva DN, Rossi M, Moran-Gilad J, Santos S, et al. chewBBACA: A complete suite for gene-by-gene schema creation and strain identification. Microb Genom. 2018;4(3):e000166. doi: 10.1099/mgen.0.000166 29543149 PMC5885018

[46] Francisco AP, Vaz C, Monteiro PT, Melo-Cristino J, Ramirez M, Carriço JA. PHYLOViZ: phylogenetic inference and data visualization for sequence based typing methods. BMC Bioinformatics. 2012;13:87. doi: 10.1186/1471-2105-13-87 22568821 PMC3403920

[47] Letunic I, Bork P. Interactive Tree of Life (iTOL) v6: recent updates to the phylogenetic tree display and annotation tool. Nucleic Acids Res. 2024;52(W1):W78–82. doi: 10.1093/nar/gkae268 38613393 PMC11223838

[48] Arndt D, Grant JR, Marcu A, Sajed T, Pon A, Liang Y, et al. PHASTER: a better, faster version of the PHAST phage search tool. Nucleic Acids Res. 2016;44(W1):W16-21. doi: 10.1093/nar/gkw387 27141966 PMC4987931

[49] Couvin D, Bernheim A, Toffano-Nioche C, Touchon M, Michalik J, Néron B, et al. CRISPRCasFinder, an update of CRISRFinder, includes a portable version, enhanced performance and integrates search for Cas proteins. Nucleic Acids Res. 2018;46(W1):W246–51. doi: 10.1093/nar/gky425 29790974 PMC6030898

[50] Cantalapiedra CP, Hernández-Plaza A, Letunic I, Bork P, Huerta-Cepas J. eggNOG-mapper v2: Functional Annotation, Orthology Assignments, and Domain Prediction at the Metagenomic Scale. Mol Biol Evol. 2021;38(12):5825–9. doi: 10.1093/molbev/msab293 34597405 PMC8662613

[51] Altschul SF, Gish W, Miller W, Myers EW, Lipman DJ. Basic local alignment search tool. J Mol Biol. 1990;215(3):403–10. doi: 10.1016/S0022-2836(05)80360-2 2231712

[52] Florensa AF, Kaas RS, Clausen PTLC, Aytan-Aktug D, Aarestrup FM. ResFinder - an open online resource for identification of antimicrobial resistance genes in next-generation sequencing data and prediction of phenotypes from genotypes. Microb Genom. 2022;8(1):000748. doi: 10.1099/mgen.0.000748 35072601 PMC8914360

[53] McArthur AG, Waglechner N, Nizam F, Yan A, Azad MA, Baylay AJ, et al. The comprehensive antibiotic resistance database. Antimicrob Agents Chemother. 2013;57(7):3348–57. doi: 10.1128/AAC.00419-13 23650175 PMC3697360

[54] Feldgarden M, Brover V, Gonzalez-Escalona N, Frye JG, Haendiges J, Haft DH, et al. AMRFinderPlus and the Reference Gene Catalog facilitate examination of the genomic links among antimicrobial resistance, stress response, and virulence. Sci Rep. 2021;11(1):12728. doi: 10.1038/s41598-021-91456-0 34135355 PMC8208984

[55] Cosentino S, Voldby Larsen M, Møller Aarestrup F, Lund O. PathogenFinder--distinguishing friend from foe using bacterial whole genome sequence data. PLoS One. 2013;8(10):e77302. doi: 10.1371/journal.pone.0077302 24204795 PMC3810466

[56] van Heel AJ, de Jong A, Song C, Viel JH, Kok J, Kuipers OP. BAGEL4: a user-friendly web server to thoroughly mine RiPPs and bacteriocins. Nucleic Acids Res. 2018;46(W1):W278–81. doi: 10.1093/nar/gky383 29788290 PMC6030817

[57] Zhou S, Liu B, Zheng D, Chen L, Yang J. VFDB 2025: an integrated resource for exploring anti-virulence compounds. Nucleic Acids Res. 2025;53(D1):D871–7. doi: 10.1093/nar/gkae968 39470738 PMC11701737

[58] Neal-McKinney JM, Liu KC, Lock CM, Wu W-H, Hu J. Comparison of MiSeq, MinION, and hybrid genome sequencing for analysis of *Campylobacter jejuni*. Sci Rep. 2021;11(1):5676. doi: 10.1038/s41598-021-84956-6 33707610 PMC7952698

[59] Wick RR, Judd LM, Holt KE. Assembling the perfect bacterial genome using Oxford Nanopore and Illumina sequencing. PLoS Comput Biol. 2023;19(3):e1010905. doi: 10.1371/journal.pcbi.1010905 36862631 PMC9980784

[60] Kastanis GJ, Santana‐Quintero LV, Sanchez‐Leon M, Lomonaco S, Brown EW, Allard MW. In‐depth comparative analysis of Illumina® MiSeq run metrics: Development of a wet‐lab quality assessment tool. Molecular Ecology Resources. 2019;19(2):377–87. doi: 10.1111/1755-0998.12973 30506954 PMC6487961

[61] Moss EL, Maghini DG, Bhatt AS. Complete, closed bacterial genomes from microbiomes using nanopore sequencing. Nat Biotechnol. 2020;38(6):701–7. doi: 10.1038/s41587-020-0422-6 32042169 PMC7283042

[62] de Souza LM, de Oliveira ID, Sales FCS, da Costa AC, Campos KR, Abbud A, et al. Technical comparison of MinIon and Illumina technologies for genotyping Chikungunya virus in clinical samples. J Genet Eng Biotechnol. 2023;21(1):88. doi: 10.1186/s43141-023-00536-3 37642827 PMC10465416

[63] Riesco R, Trujillo ME. Update on the proposed minimal standards for the use of genome data for the taxonomy of prokaryotes. Int J Syst Evol Microbiol. 2024;74(3):006300. doi: 10.1099/ijsem.0.006300 38512750 PMC10963913

[64] Ceapa C, Davids M, Ritari J, Lambert J, Wels M, Douillard FP, et al. The Variable Regions of *Lactobacillus rhamnosus* Genomes Reveal the Dynamic Evolution of Metabolic and Host-Adaptation Repertoires. Genome Biol Evol. 2016;8(6):1889–905. doi: 10.1093/gbe/evw123 27358423 PMC4943194

[65] Rodriguez-R LM, Conrad RE, Viver T, Feistel DJ, Lindner BG, Venter SN, et al. An ANI gap within bacterial species that advances the definitions of intra-species units. mBio. 2024;15(1):e0269623. doi: 10.1128/mbio.02696-23 38085031 PMC10790751

[66] Maiden MCJ, Jansen van Rensburg MJ, Bray JE, Earle SG, Ford SA, Jolley KA, et al. MLST revisited: the gene-by-gene approach to bacterial genomics. Nat Rev Microbiol. 2013;11(10):728–36. doi: 10.1038/nrmicro3093 23979428 PMC3980634

[67] de Sales RO, Migliorini LB, Puga R, Kocsis B, Severino P. A Core Genome Multilocus Sequence Typing Scheme for *Pseudomonas aeruginosa*. Front Microbiol. 2020;11:1049. doi: 10.3389/fmicb.2020.01049 32528447 PMC7264379

[68] Jarocki P, Komoń-Janczara E, Młodzińska A, Sadurski J, Kołodzińska K, Łaczmański Ł, et al. Occurrence and genetic diversity of prophage sequences identified in the genomes of *L. casei* group bacteria. Sci Rep. 2023;13(1):8603. doi: 10.1038/s41598-023-35823-z 37237003 PMC10219966

[69] Mills S, Shanahan F, Stanton C, Hill C, Coffey A, Ross RP. Movers and shakers: influence of bacteriophages in shaping the mammalian gut microbiota. Gut Microbes. 2013;4(1):4–16. doi: 10.4161/gmic.22371 23022738 PMC3555884

[70] de Jonge PA, Wortelboer K, Scheithauer TPM, van den Born B-JH, Zwinderman AH, Nobrega FL, et al. Gut virome profiling identifies a widespread bacteriophage family associated with metabolic syndrome. Nat Commun. 2022;13(1):3594. doi: 10.1038/s41467-022-31390-5 35739117 PMC9226167

[71] Nanda AM, Thormann K, Frunzke J. Impact of spontaneous prophage induction on the fitness of bacterial populations and host-microbe interactions. J Bacteriol. 2015;197(3):410–9. doi: 10.1128/JB.02230-14 25404701 PMC4285972

[72] Pei Z, Sadiq FA, Han X, Zhao J, Zhang H, Ross RP, et al. Comprehensive Scanning of Prophages in *Lactobacillus*: Distribution, Diversity, Antibiotic Resistance Genes, and Linkages with CRISPR-Cas Systems. mSystems. 2021;6(3):e0121120. doi: 10.1128/mSystems.01211-20 34060909 PMC8269257

[73] Li Q. CRISPR Typing of Salmonella Isolates from Different Resources. Methods Mol Biol. 2021;2182:45–50. doi: 10.1007/978-1-0716-0791-6_6 32894486

[74] Shariat N, Dudley EG. CRISPRs: molecular signatures used for pathogen subtyping. Appl Environ Microbiol. 2014;80(2):430–9. doi: 10.1128/AEM.02790-13 24162568 PMC3911090

[75] Pan H, Jia C, Paudyal N, Li F, Mao J, Liu X, et al. Comprehensive Assessment of Subtyping Methods for Improved Surveillance of Foodborne Salmonella. Microbiol Spectr. 2022;10(5):e0247922. doi: 10.1128/spectrum.02479-22 36194132 PMC9602514

[76] Kunin V, Sorek R, Hugenholtz P. Evolutionary conservation of sequence and secondary structures in CRISPR repeats. Genome Biol. 2007;8(4):R61. doi: 10.1186/gb-2007-8-4-r61 17442114 PMC1896005

[77] Tisza MJ, Buck CB. A catalog of tens of thousands of viruses from human metagenomes reveals hidden associations with chronic diseases. Proc Natl Acad Sci U S A. 2021;118(23):e2023202118. doi: 10.1073/pnas.2023202118 34083435 PMC8201803

[78] Durmaz E, Miller MJ, Azcarate-Peril MA, Toon SP, Klaenhammer TR. Genome sequence and characteristics of Lrm1, a prophage from industrial Lactobacillus rhamnosus strain M1. Appl Environ Microbiol. 2008;74(15):4601–9. doi: 10.1128/AEM.00010-08 18539811 PMC2519341

[79] Jarocki P, Komoń-Janczara E, Podleśny M, Kholiavskyi O, Pytka M, Kordowska-Wiater M. Genomic and Proteomic Characterization of Bacteriophage BH1 Spontaneously Released from Probiotic *Lactobacillus rhamnosus* Pen. Viruses. 2019;11(12):1163. doi: 10.3390/v11121163 31888239 PMC6950654

[80] Quistgaard EM, Löw C, Guettou F, Nordlund P. Understanding transport by the major facilitator superfamily (MFS): structures pave the way. Nat Rev Mol Cell Biol. 2016;17(2):123–32. doi: 10.1038/nrm.2015.25 26758938

[81] Lekshmi M, Ortiz-Alegria A, Kumar S, Varela MF. Major facilitator superfamily efflux pumps in human pathogens: Role in multidrug resistance and beyond. Curr Res Microb Sci. 2024;7:100248. doi: 10.1016/j.crmicr.2024.100248 38974671 PMC11225705

[82] Nadkarni MA, Chen Z, Wilkins MR, Hunter N. Comparative genome analysis of *Lactobacillus rhamnosus* clinical isolates from initial stages of dental pulp infection: identification of a new exopolysaccharide cluster. PLoS One. 2014;9(3):e90643. doi: 10.1371/journal.pone.0090643 24632842 PMC3954586

[83] von Ossowski I, Satokari R, Reunanen J, Lebeer S, De Keersmaecker SCJ, Vanderleyden J, et al. Functional characterization of a mucus-specific LPXTG surface adhesin from probiotic *Lactobacillus rhamnosus* GG. Appl Environ Microbiol. 2011;77(13):4465–72. doi: 10.1128/AEM.02497-10 21602388 PMC3127685

[84] Spirig T, Weiner EM, Clubb RT. Sortase enzymes in Gram-positive bacteria. Mol Microbiol. 2011;82(5):1044–59. doi: 10.1111/j.1365-2958.2011.07887.x 22026821 PMC3590066

[85] Cascioferro S, Totsika M, Schillaci D. Sortase A: an ideal target for anti-virulence drug development. Microb Pathog. 2014;77:105–12. doi: 10.1016/j.micpath.2014.10.007 25457798

[86] Susmitha A, Bajaj H, Madhavan Nampoothiri K. The divergent roles of sortase in the biology of Gram-positive bacteria. Cell Surf. 2021;7:100055. doi: 10.1016/j.tcsw.2021.100055 34195501 PMC8225981

[87] von Ossowski I, Reunanen J, Satokari R, Vesterlund S, Kankainen M, Huhtinen H, et al. Mucosal adhesion properties of the probiotic *Lactobacillus rhamnosus* GG SpaCBA and SpaFED pilin subunits. Appl Environ Microbiol. 2010;76(7):2049–57. doi: 10.1128/AEM.01958-09 20118368 PMC2849237

[88] Rintahaka J, Yu X, Kant R, Palva A, von Ossowski I. Phenotypical analysis of the *Lactobacillus rhamnosus* GG fimbrial spaFED operon: surface expression and functional characterization of recombinant SpaFED pili in Lactococcus lactis. PLoS One. 2014;9(11):e113922. doi: 10.1371/journal.pone.0113922 25415357 PMC4240662

[89] Koryszewska-Bagińska A, Gawor J, Nowak A, Grynberg M, Aleksandrzak-Piekarczyk T. Comparative genomics and functional analysis of a highly adhesive dairy *Lactobacillus paracasei* subsp. paracasei IBB3423 strain. Appl Microbiol Biotechnol. 2019;103(18):7617–34. doi: 10.1007/s00253-019-10010-1 31359102 PMC6717177

[90] Wu D, Dai M, Shi Y, Zhou Q, Li P, Gu Q. Purification and characterization of bacteriocin produced by a strain of *Lacticaseibacillus rhamnosus* ZFM216. Front Microbiol. 2022;13:1050807. doi: 10.3389/fmicb.2022.1050807 36439838 PMC9684204

[91] Zheng J, Gänzle MG, Lin XB, Ruan L, Sun M. Diversity and dynamics of bacteriocins from human microbiome. Environ Microbiol. 2015;17(6):2133–43. doi: 10.1111/1462-2920.12662 25346017

[92] Wonglapsuwan M, Pahumunto N, Teanpaisan R, Surachat K. Unlocking the genetic potential of *Lacticaseibacillus rhamnosus* strains: Medical applications of a promising probiotic for human and animal health. Heliyon. 2024;10(8):e29499. doi: 10.1016/j.heliyon.2024.e29499 38655288 PMC11035056

