# Supplementary material for: Genomic characteristics of Lacticaseibacillus rhamnosus strains isolated from blood
Source: PLoS One. 2025 Oct 31;20(10):e0335843. doi: 10.1371/journal.pone.0335843 (PMC12578247; doi:10.1371/journal.pone.0335843)
Supplement: S3 Fig — Distances were calculated with cgmlst/dists, and the MST was generated using NetworkX (Kruskal’s algorithm). The resulting tree was exported in Newick format and visualized with iTOL. Concentric circles around strains indicate metadata: the inner circle shows geographic origin, and the outer circle shows the isolation source, both encoded with colors as specified in the legend. Strains analyzed in this study are highlighted in green. (PDF) [file pone.0335843.s003.pdf]

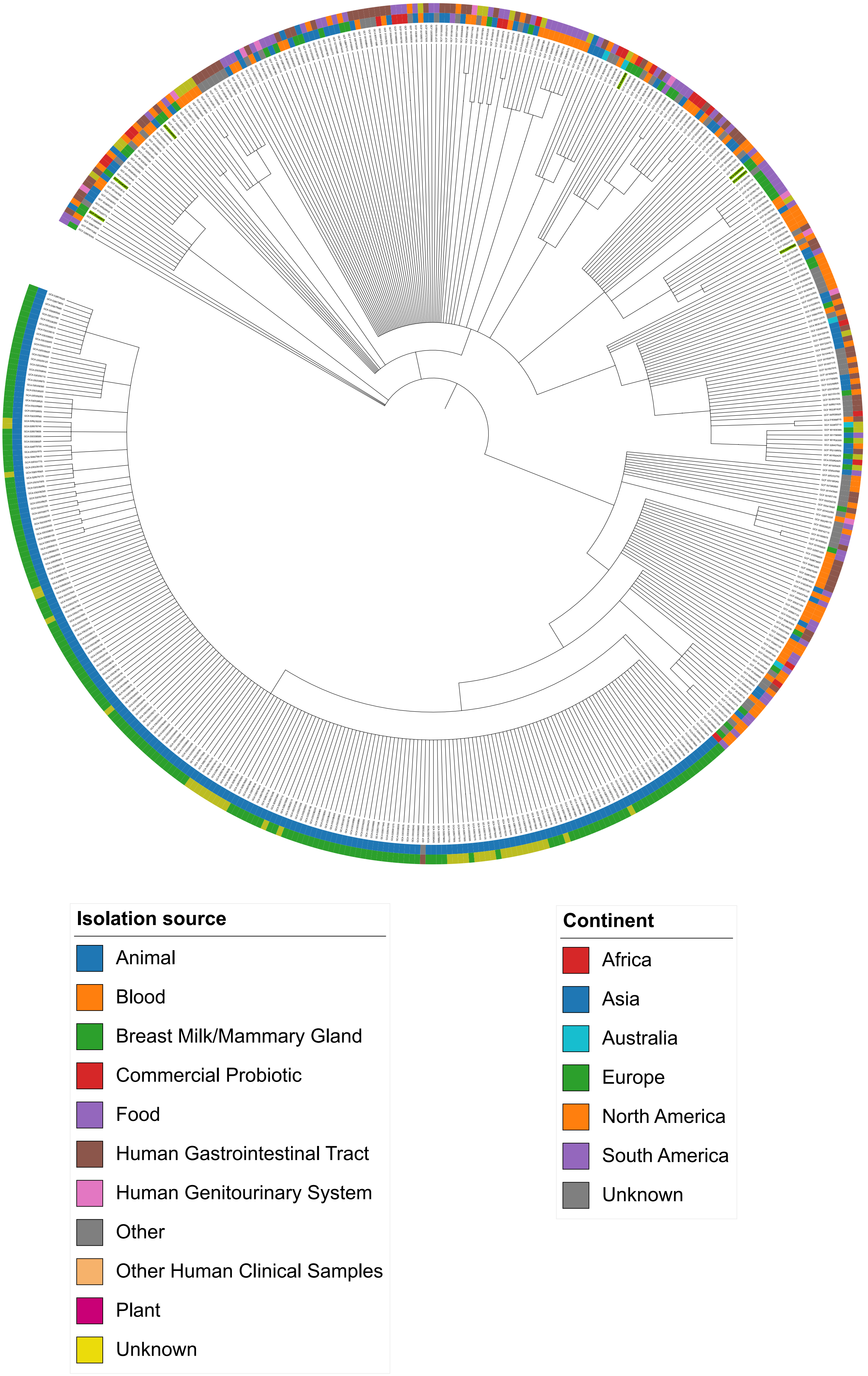
Isolation source
Animal
Blood
Breast Milk/Mammary Gland
Commercial Probiotic
Food
Human Gastrointestinal Tract
Human Genitourinary System
Other
Other Human Clinical Samples
Plant
Unknown
Continent
Africa
Asia
Australia
Europe
North America
South America
Unknown
